# A Clathrin light chain A reporter mouse for *in vivo* imaging of endocytosis

**DOI:** 10.1371/journal.pone.0273660

**Published:** 2022-09-23

**Authors:** Elisabeth Grimm, Franciscus van der Hoeven, Donato Sardella, Katrin I. Willig, Ulrike Engel, Nisha Veits, Robert Engel, Elisabetta Ada Cavalcanti-Adam, Felix Bestvater, Luca Bordoni, Richard Jennemann, Kai Schönig, Ina Maria Schiessl, Roger Sandhoff

**Affiliations:** 1 Lipid Pathobiochemistry Group, German Cancer Research Center (DKFZ), Heidelberg, Germany; 2 Faculty of Biosciences, Heidelberg University, Heidelberg, Germany; 3 Transgenic Service, German Cancer Research Center (DKFZ), Heidelberg, Germany; 4 Department of Biomedicine, Aarhus University, Aarhus, Denmark; 5 Optical Nanoscopy in Neuroscience, Center for Nanoscale Microscopy and Molecular Physiology of the Brain, University Medical Center Göttingen, Göttingen, Germany; 6 Max Planck Institute of Experimental Medicine, Goettingen, Germany; 7 Nikon Imaging Center at Heidelberg University and Centre of Organismal Studies (COS), Bioquant, Heidelberg, Germany; 8 Department of Cellular Biophysics, Max Planck Institute for Medical Research, Heidelberg, Germany; 9 Light Microscopy Core Facility, German Cancer Research Center (DKFZ), Heidelberg, Germany; 10 Department of Molecular Biology, Central Institute of Mental Health, Mannheim, Germany; Institut Curie, FRANCE

## Abstract

Clathrin-mediated endocytosis (CME) is one of the best studied cellular uptake pathways and its contributions to nutrient uptake, receptor signaling, and maintenance of the lipid membrane homeostasis have been already elucidated. Today, we still have a lack of understanding how the different components of this pathway cooperate dynamically *in vivo*. Therefore, we generated a reporter mouse model for CME by fusing eGFP endogenously in frame to clathrin light chain a (Clta) to track endocytosis in living mice. The fusion protein is expressed in all tissues, but in a cell specific manner, and can be visualized using fluorescence microscopy. Recruitment to nanobeads recorded by TIRF microscopy validated the functionality of the Clta-eGFP reporter. With this reporter model we were able to track the dynamics of Alexa594-BSA uptake in kidneys of anesthetized mice using intravital 2-photon microscopy. This reporter mouse model is not only a suitable and powerful tool to track CME *in vivo* in genetic or disease mouse models it can also help to shed light into the differential roles of the two clathrin light chain isoforms in health and disease.

## Introduction

### Technical background

The use of animal models is essential to study gene function, developmental processes, and (human) diseases [1, 2]. In 2013, a new method to generate gene-modified animals, named clustered regulatory interspaced short palindromic repeat/CRISPR-associated protein 9 (CRISPR/Cas9) was reported [3, 4]. Previously, it was time consuming to generate gene-modified animals by using zinc-finger nucleases (ZFNs), transcription activator-like effector nucleases (TALENs) or conventional stem cell targeting [5]. The CRISPR/Cas9 system is an adaptive immune system in bacteria for the detection and destruction of invading viruses and plasmids [6]. This system can be used to insert sequence-specific double-strand breaks (DSB) in a zygote or cells by providing Cas9 and either a sgRNA (single guide RNA) or crRNA (CRISPR RNA) and tracrRNA (transactivating crRNA) together for the generation of a gene knock-out (KO) or a knock-in (KI) through insertion or deletion mutations (indel) via non-homogenous end joining or homologous directed-repair (HDR) by providing a DNA repair template, respectively [7]. Since the initial CRISPR/Cas9 description as an easy and fast to use approach for genomic engineering appeared, various publications with optimized protocols for this system reported a good specificity and selectivity for the reported loci [8–12].

### Biological context

The physiological process for the uptake of molecules into the cell is highly evolutionary conserved as well as essential to higher eukaryotic life [13, 14]. There are different routes described, but the best characterized pathway is clathrin-mediated endocytosis (CME), which is involved in nutrient uptake, regulation of cell signalling, cell adhesion and maintenance of the plasma membrane compositional homeostasis [15, 16]. The pathway is named after the key cytosolic component of this endocytic machinery, the clathrin protein. These proteins form a triskelion, composed of three clathrin heavy chain proteins (CHC) and three clathrin light chain proteins (Lc) [17]. There are two clathrin light chain isoforms, clathrin light chain a and b (Clta or Cltb) known by now [18]. This highly coordinated and complex uptake pathway requires the formation of a clathrin-coated pit (CCP) at the plasma membrane, which is then internalized into the cell as a clathrin-coated vesicle (CCV). The different steps of CME are partially overlapping, starting with the initiation of the CCP at the plasma membrane with subsequent cargo selection and recruitment as well as the further recruitment of endocytic proteins from the cytosolic pool. After membrane curvature induction the CCP scission, which is dependent on the GTPase dynamin, led to the separation of the CCV from the plasma membrane. The CCV is then routed towards the cytosol, where uncoating takes place and the vesicle is fused with the early endosome. The cargo is either degraded or recycled to the plasma membrane [19]. Even if CME is the best characterized uptake mechanism, there is still a lack of understanding of how the different components work dynamically in multicellular organisms (*in vivo*) [20]. Especially the differential roles of Clta and Cltb in health and disease is under investigation [21–23]. The classical techniques to study CME are various cell-free and cell-based assays as well as 3D culture systems [24–26]. These (cell culture) systems help to understand the basics of CME, but there are still limitations like induced physiological changes by the unlimited availability of nutrients and growth factors and more important the dynamics cannot be investigated *in vivo* with these systems [27, 28].

### Subject of this work

Here we describe the generation and characterization of a new CME reporter mouse model, Clta^em1(GFP)Rsnd^, for the use with intravital microscopy by using the CRISPR/Cas9 method. Therefore, we introduced eGFP in-frame at the endogenous C-terminus of the Clta locus.

## Results

### Generation of the Clta-eGFP-KI mouse using CRIPSR/Cas9

**Fig 1:**
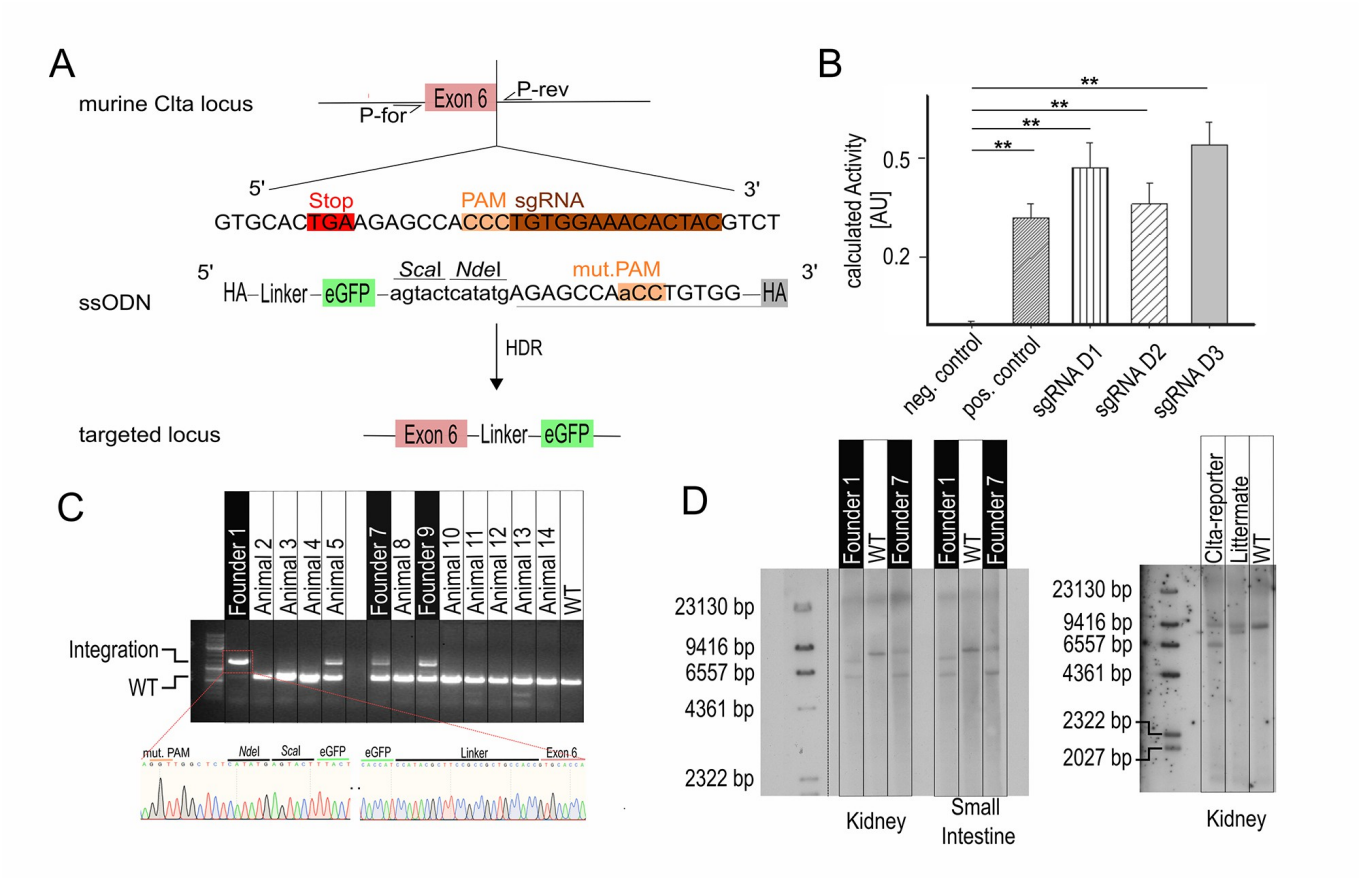
Endogenous tagging of Clathrin light chain a. (**A**) Targeting strategy for C-terminally endogenous tagging of Clta with eGFP using CRISPR/Cas9. The eGFP is fused in-frame to Clta separated by a flexible linker. Additional restriction recognition sites were introduced for screening. The original PAM was mutated to prevent re-cleaving of the inserted DNA. P-for/P-rev: annealing sites of forward and reverse primers for genotyping, HA: homology arm (5’ 115 bp without stop codon, 3’ 98 bp). (**B**) Cleaving activity of the selected gRNAs using a gene complementation assay. Representative data of two individual experiments is shown. n = 3 transfections; error bars: SD; **: p<0.01 (students t-test). (**C**) Genotyping and sequencing of 14 live-born animals after pro-nuclear injection of crRNA-tracrRNA complex, Cas9 mRNA and ssDNA repair template. Fragment size WT: 591 bp and Clta-reporter: 1349 bp. Forward primer binding site on deleted allele of founder 1 missing, therefore no amplification. (**D**) Southern Blot Analysis of founder 1, founder 7 and WT in kidney and small intestine. Founder 1 shows, in addition to the integration band (expected at 6130 bp), a band at ~7500 bp, which is indicative for a deletion. Founder 7 shows a band migrating at the WT size (expected at 8444 bp) and one smaller band (about 6130 bp) indicative for the integration (**left**). Offsprings of F1-generation of founder 1 showed animals harboring the WT-Clta allele and an integration allele (Clta-reporter) and animals harboring the WT allele and the deletion allele (littermate) (**right**).

Here we report the use of the CRIPSR/Cas9 system to generate a reporter KI mouse for CME. Therefore, the cDNA of eGFP was fused endogenously to the C-terminus of Clta separated by a GGSGSVWV-linker (Fig 1A). This linker was previously used by Anderson et al. to generate a Clta-KI in hiPSC and we decided to use this linker, as Clta was shown to be functional [29]. The C-terminal tagging has been described to have no adverse effects on the protein function [30, 31]. Apart from that, this endogenous targeting strategy was used to circumvent overexpression artefacts as an increased CCP lifetime was reported in cells overexpressing a fluorescently tagged Clta compared to cells expressing the Clta-fusion protein at endogenous levels [30]. Furthermore, it was seen in yeast that stoichiometry is important in endocytic processes [32]. Using CRISPOR.tefor.net [33, 34] three individual single guide sequences with high specificity scores were selected to target the stop codon of the Clta gene. The three sgRNA sequences were cloned into the pX330 vector and their capability of cleaving the genomic region of Clta was validated using a gene-complementation assay in HeLa cells (Fig 1B) [35]. The sgRNA that showed the best *in vitro* cutting efficiency was used for the targeting. Furthermore, Anderson *et al*., used in their targeting approach the same sgRNA, that showed the best *in vitro* cutting efficiency in our screen. To achieve HDR a ssDNA repair-template as described by Miura *et al*., was used, as it was reported that ssDNA repair templates show higher frequency of HDR [8]. Therefore, the targeting region was flanked by 98 bp and 115 bp homology arms up- and downstream of the targeting site. The validated sgRNA was injected as annealed crRNA-tracrRNA complex together with the ssDNA repair template and Cas9-protein pro-nuclear into zygotes of C57BL/6N mice. A single point mutation (C→A) was introduced into the PAM sequence of the ssDNA repair template to prevent re-cutting of the inserted sequence (see Fig 1A). Genomic PCR of all 14 live born animals revealed 4 targeted animals, sequencing in turn showed correct sequence in 3 out of 4 animals (Fig 1C). In addition, the genotypes were confirmed using Southern Blot analysis (Fig 1D). All 4 animals were heterozygous for the integration. Using Southern Blot analysis and sequencing an 855 bp deletion downstream of the sgRNA-mediated cut site was detected in animal 1 (Fig 1D left) and half of its offsprings (Fig 1D right and S1A Fig in S1 Appendix). Animal 5 showed two point-mutations, resulting in amino acid exchanges (Thr98Asn and Leu232Gln) in the eGFP sequence (S1B Fig in S1 Appendix). Germline transmission from F0 generation to F1 generation was seen in all founder lines. In addition, those off targets, predicted by CRIPSPOR.tefor.net, that have a high cutting frequency determination (CFD) score, off-targets within an exon and off-targets on chromosome 4 were selected and tested using PCR and subsequent sequencing. None of the tested off-targets were edited (S1C Fig in S1 Appendix) and breeding to C57BL/6N animals further dilutes off target occurrence. During breeding no phenotypic and morphologic changes (S2 Fig in S1 Appendix) and no further phenotype (e.g. no behavioural changes and no reproductive abnormalities) were observed in heterozygous bred animals.

### Expression of the Clta-eGFP fusion protein

**Fig 2:**
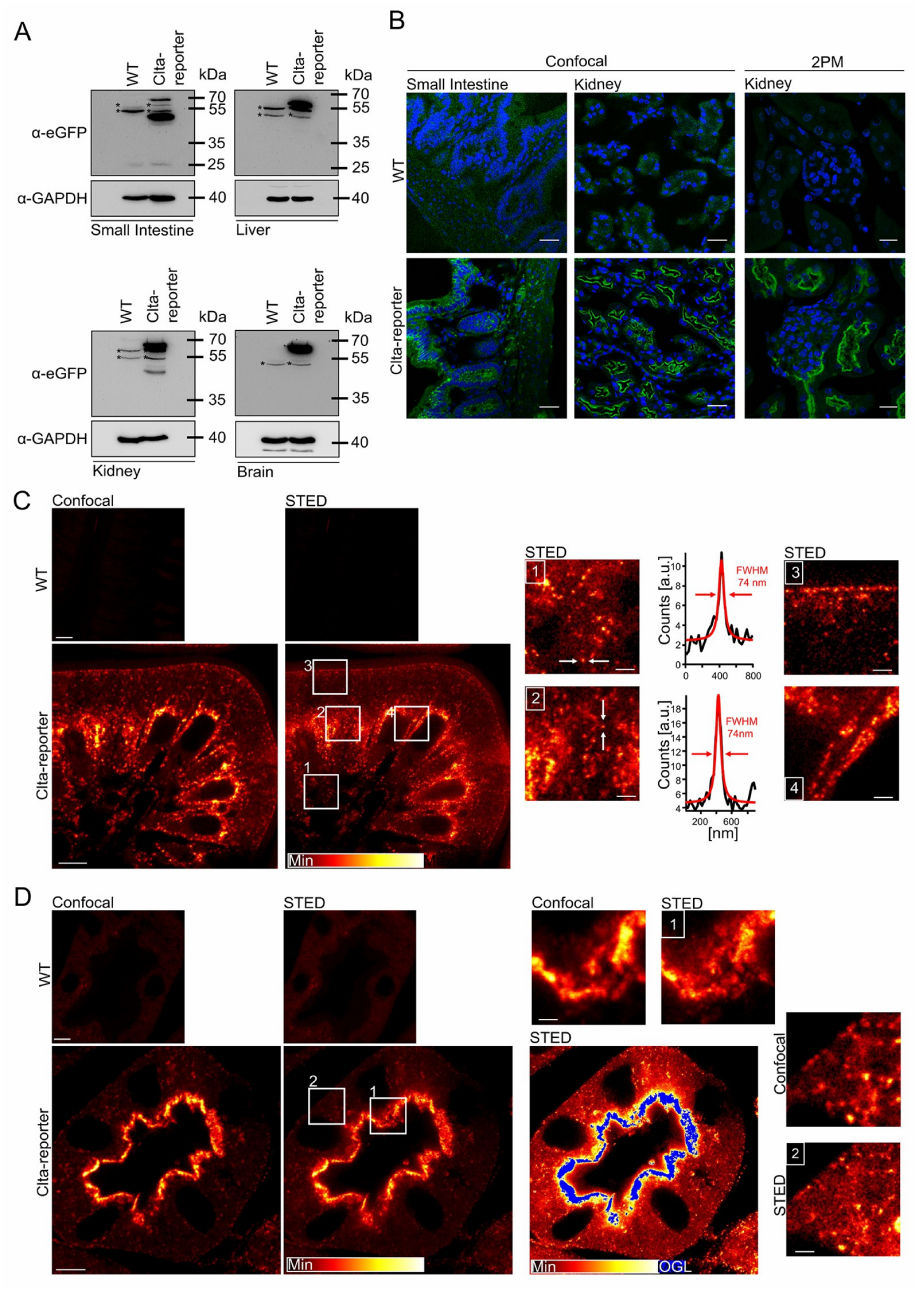
Tissue expression of Clta-eGFP fusion protein. (**A**) Western Blot Analysis of small intestine, brain, kidney and liver lysates show the expression of the Clta-eGFP fusion protein. Additional band in small intestine and kidney arises possibly through tissue specifc splicing or exon-skipping through insertion of eGFP. Partial degradation may not be excluded. On each lane 25 μg of total protein were loaded. *: unspecific cross reactivity of the eGFP antibody. (**B**) Immunofluorescence of dye separated WT and Clta-reporter cryo sections of kidney and small intestine using confocal microscopy (**left**, scale bar 30 μm) and 2-PM (**right**, scale bar 15 μm). The related WT emission spectra in the green spectral range recorded with confocal microscopy, indicating an autofluoresence maxima at 550 nm can be found in S3B Fig in S1 Appendix. (**C**) Confocal and associated STED images of small intestine of WT and Clta-reporter cryo sections revealed a strong apical occurrence. Scale bar: 5 μm and 1 μm for the cut-outs (1–4). Clta-eGFP appears in clusters of 74 nm in size or smaller. (**D**) Confocal and associated STED images of kidney of WT and Clta-reporter cryo sections. A strong apical localization of the Clta-eGFP fusion protein was detected. Scale bar: 5 μm and 1 μm for the cutouts (1–2). Darker regions in the STED image are shown by a saturated color table with overglow (OGL) in blue.

The expression of the Clta-eGFP fusion protein was confirmed by Western Blot analysis of whole lysates of brain, lung, heart, spleen, liver, kidney, and small intestine. The fusion protein was detected in all tissues examined using an anti-eGFP antibody (Fig 2A and S3A Fig in S1 Appendix). The protein band was detected at the expected size of the Clta-eGFP fusion protein of about 57 kDa except for small intestine and kidney, where in addition a band of about 40 kDa was detected (Fig 2A). The presence of the Clta-eGFP fusion protein was further confirmed by confocal fluorescence microscopy, 2-PM and superresolution stimulated emission depletion (STED) microscopy of cryosections (Fig 2B–2D). The fluorescence signal was detected in all tissues examined, however a dye-separation approach (see material and methods section) helped to distinguish the autofluorescence from the Clta-eGFP signal in liver, heart, lung and spleen (Fig 2B and S3C-S3F Fig in S1 Appendix), as tissue sections are known to show autofluorescence in green and blue spectral range [35]. Highly polarized tissue as small intestine and kidney showed a strong apical localization of the Clta-eGFP fusion protein (Fig 2B). Using STED microscopy, we resolved a clustered distribution of Clta-eGFP in these organs (Fig 2C and 2D).

**Fig 3:**
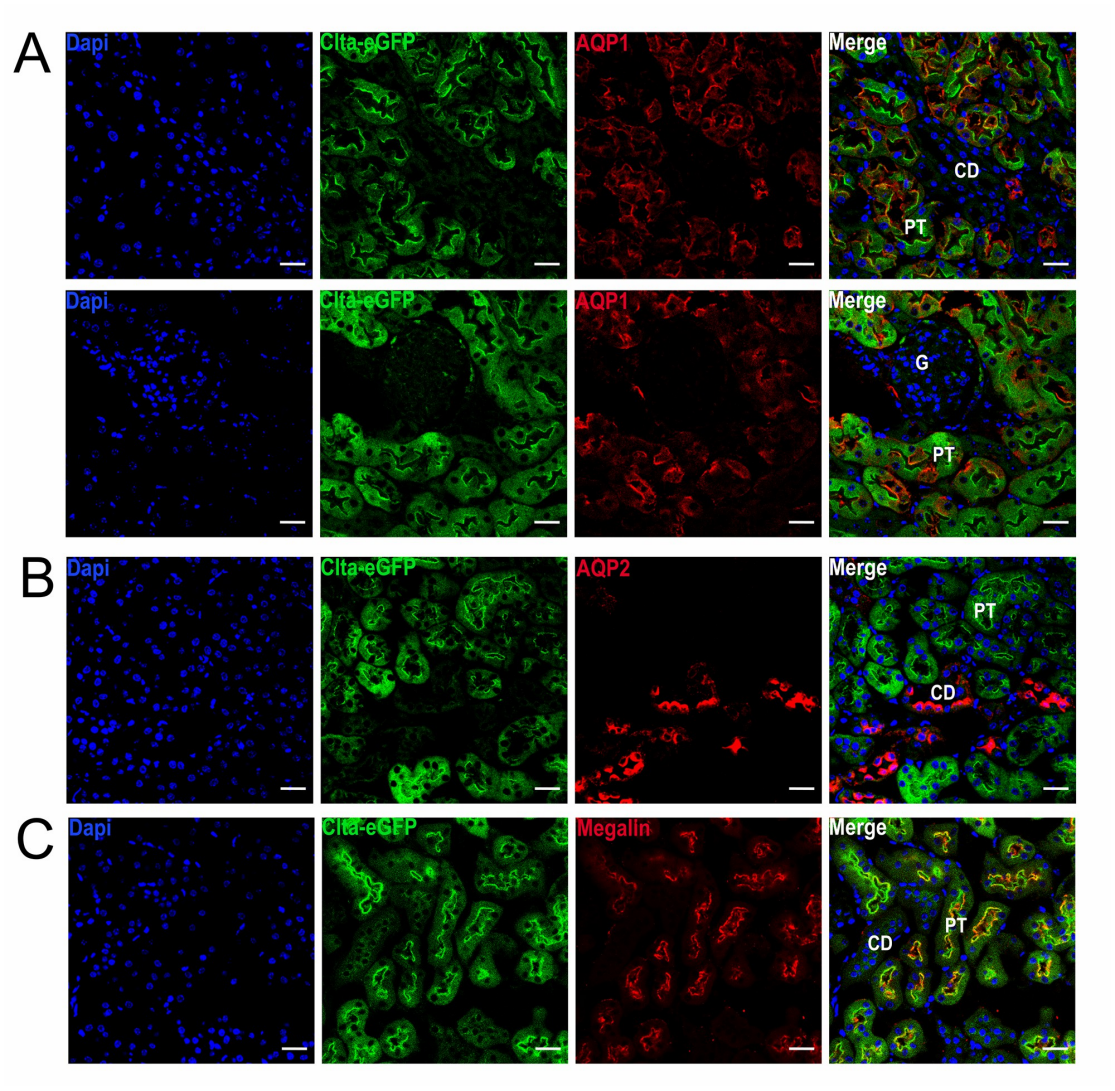
Localization of the Clta-eGFP signal in kidney sections. (**A**) Clta-eGFP reporter kidney section stained for aquaporin-1 (AQP1) showing that the Clta-eGFP signal is localized to proximal tubule. (**B**) Clta-eGFP reporter kidney sections stained for aquaporin-2 (AQP2) showing that the Clta-eGFP is not detectable in collecting duct of murine kidney. (**C**) Clta-eGFP reporter co-localizes partly with the brush border resident protein megalin. Scale bar: 25 μm, CD: collecting duct, G: glomerulus, PT: proximal tubule.

The Clta-eGFP fusion protein is expressed in proximal tubules (Fig 3A) whereas other areas of the kidney like the collecting duct are negative for Clta-eGFP expression (Fig 3B) [36]. Furthermore, it partly co-localizes with the brush border resident protein megalin (Fig 3C). The interaction of the CCP and megalin occurs during CME and apical trafficking of the receptor [37–39].

### Monitoring the Clta-eGFP fusion protein in primary fibroblasts

**Fig 4:**
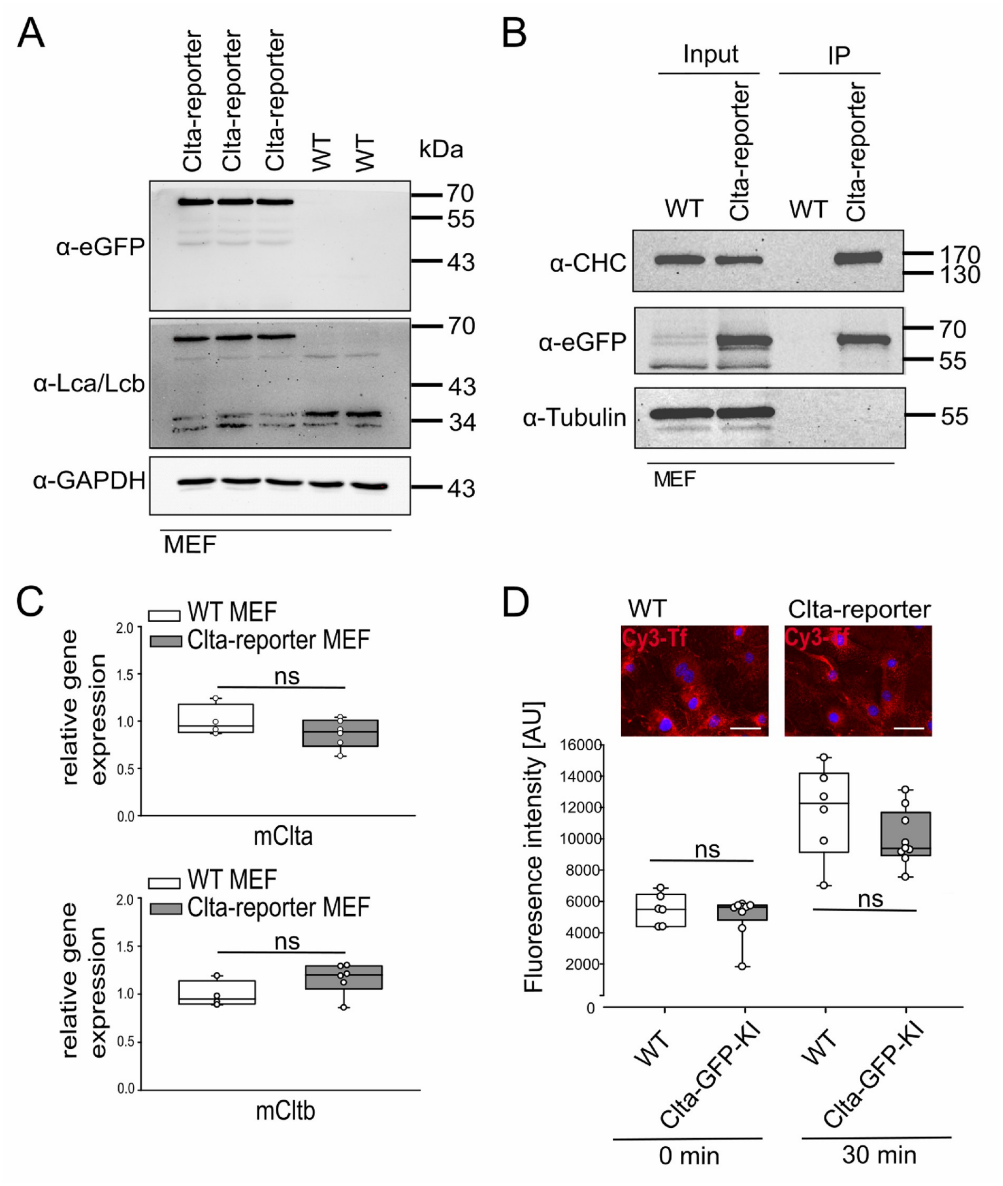
Clta-eGFP in primary MEFs. (**A**) Western blot of three Clta-reporter MEF and two WT MEF cell lines. Clta-eGFP fusion protein is detected using anti-eGFP and anti-Lc antibodies. On each lane 20 μg of total protein were loaded. (**B**) Co-IP of CHC and Clta-eGFP fusion protein in MEFs. (**C**) qPCR of Clta and Cltb in MEFs. No significant changes were observed in Clta-reporter and WT MEFs. WT: n = 4; Clta-reporter n = 6 (students t-test). (**D**) Cy3-Tf uptake in Clta-reporter and WT MEFs: no significant changes in the uptake of Cy3-Tf were detected between WT and Clta-reporter MEFs at 0 min and 30 min (students t-test). WT: n = 6; Clta-reporter n = 9; Scale bar: 50 μm.

Primary mouse embryonic fibroblasts (MEFs) were isolated from WT and Clta-reporter animals to further characterize the Clta-eGFP mouse model. Using Western Blot analysis, the expression of the Clta-eGFP fusion protein was confirmed (Fig 4A). For the formation of clathrin triskelia which are used to build CCPs at the plasma membrane three CHCs interact with three CLCs. The C-terminal part of the CLC is located at the vertex of the triskelion [40]. To test whether the C-terminal eGFP disturbs the interaction of Clta with CHC we carried out co-immunoprecipitation (Co-IP) and co-localization analyses according to Biancospino *et al*. [22]. The CHC was detected using western blotting after immunoprecipitation of Clta-eGFP fusion protein with GFP-trap magnetic agarose. This indicates that the interaction of CHC and Clta-eGFP is not altered (Fig 4B). This finding was confirmed by co-localization image analysis of CHC-Lc and CHC-Clta-eGFP staining (S4A Fig in S1 Appendix). Furthermore, qPCR was carried out to investigate whether the expression of the Clta-eGFP fusion protein had adverse effects influencing the expression of Clta or Cltb, as it was described that KO of one isoform leads to increased expression of the other isoform [23]. Comparing the expression levels of Clta and Cltb in WT MEFs and Clta-reporter MEFs no significant differences were observed (Fig 4C).

**Fig 5:**
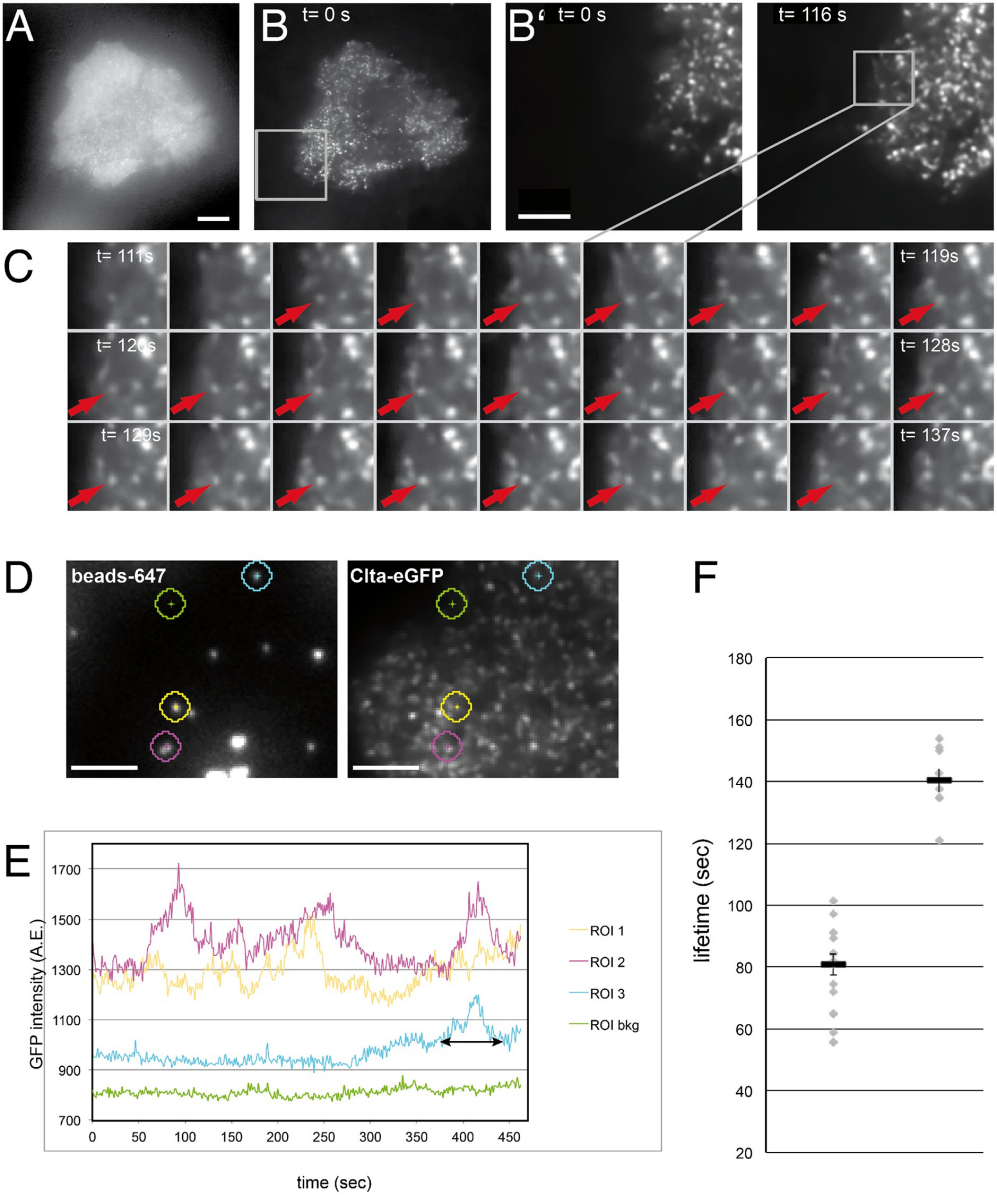
Clta-eGFP dynamics and recruitment to immobilized beads. MEFs from Clta-eGFP mice show dynamic puncta consistent with Clta-eGFP localization at coated pits and coated vesicles. (**A-C**) Clta-eGFP expressing cell imaged by wide field fluorescence (**A**) and TIRFM (**B**, **C**). The cell was imaged at 1 fps for 3 minutes. In (**B’**) the region indicated in (**B**) is magnified for the indicated times to show the temporal changes of Clta-eGFP puncta. (**C**) Time lapse of a 5 μm region indicated in B’ where a Clta-eGFP spot appears (arrow) and loses intensity before disappearing at t = 137 s. (**D-F**) Recruitment of Clta-eGFP to immobilized beads. (**D**) TIRF imaging of beads and Clta-eGFP (**E**) Representative analysis of Clta-eGFP intensity at beads (ROI 1–3) over time and in a control region (green, background bkg). Note that the regions shown in (**D**) have been enlarged for clarity, regions analyzed only comprised 12 pixels (0,41 μm^2^). The time of elevated GFP intensity is indicated as “lifetime”, e.g. as for ROI3 (cyan) where Clta-eGFP was recruited at the end of the time lapse. (**F**) Clta-eGFP lifetimes collected from 6 cells on beads. Lifetimes measured clustered around 81.0 seconds and 140.5 seconds (median of values), error bars indicate standard mean error. Scale bars are A = 10 μm, B’, D = 5 μm.

For high resolution microscopy we plated MEFs of Clta-eGFP mice on coverslip chambers and imaged cells with total internal reflection fluorescence (TIRF) microscopy (TIRFM), which results in high contrast images of structures close to the substrate (compare Fig 5A and 5B). Clta-eGFP was found in distinct puncta at the cell membrane (Fig 5B and 5C, that formed and disappeared (see also S1 Movie). When disappearing, a gradual loss of intensity in TIRFM indicated that the puncta moved away from the membrane into cytosol (Fig 5C), consistent with clathrin-coated vesicles budding off the membrane. To demonstrate that the Clta-eGFP puncta are functional clathrin coated pits, we made use of a previously established assay, where immobilized nanobeads of 100–300 nm were shown to specifically elicit the repetitive formation of clathrin coated pits and vesicles from the cell ventral side [24]. When we plated the MEFs on immobilized beads of 200 nm in size (Fig 5D and 5F) we also detected repetitive recruitment of Clta-eGFP on these beads (Fig 5E), with a typical lifetime of 81 seconds (Fig 5F), indeed very similar to the lifetimes of AP2-containing structures at 100 nm beads (88.5 +/- 22) observed by Fratini *et al*. [24]. We also detected lifetimes that clustered at 140.5 seconds. We interpret the shorter lifetime as single recruitment, while the higher lifetimes are probably overlapping multiple recruitments.

A transferrin uptake assay using Cy3-labelled holo-transferrin (Tf) was carried out to test whether the Clta-eGFP fusion protein impairs the uptake of CME-cargoes [41, 42]. Even if, Clta is not necessary for the uptake of conventional CME cargoes, this assay shows whether global CME is disrupted. No significant difference in the uptake of Cy3Tf between WT MEFs and Clta-reporter MEFs was detected (Fig 4D). Furthermore, treatment of WT and Clta-reporter MEFs with 5 μg/mL of the CME inhibitor chlorpromazine (CPZ) 15 min prior and during the Cy3Tf uptake reduced the uptake (S5 Fig in S1 Appendix).

### Visualization of in vivo albumin uptake in proximal tubular epithelial cells (PTEC) of Clta-reporter mice

**Fig 6:**
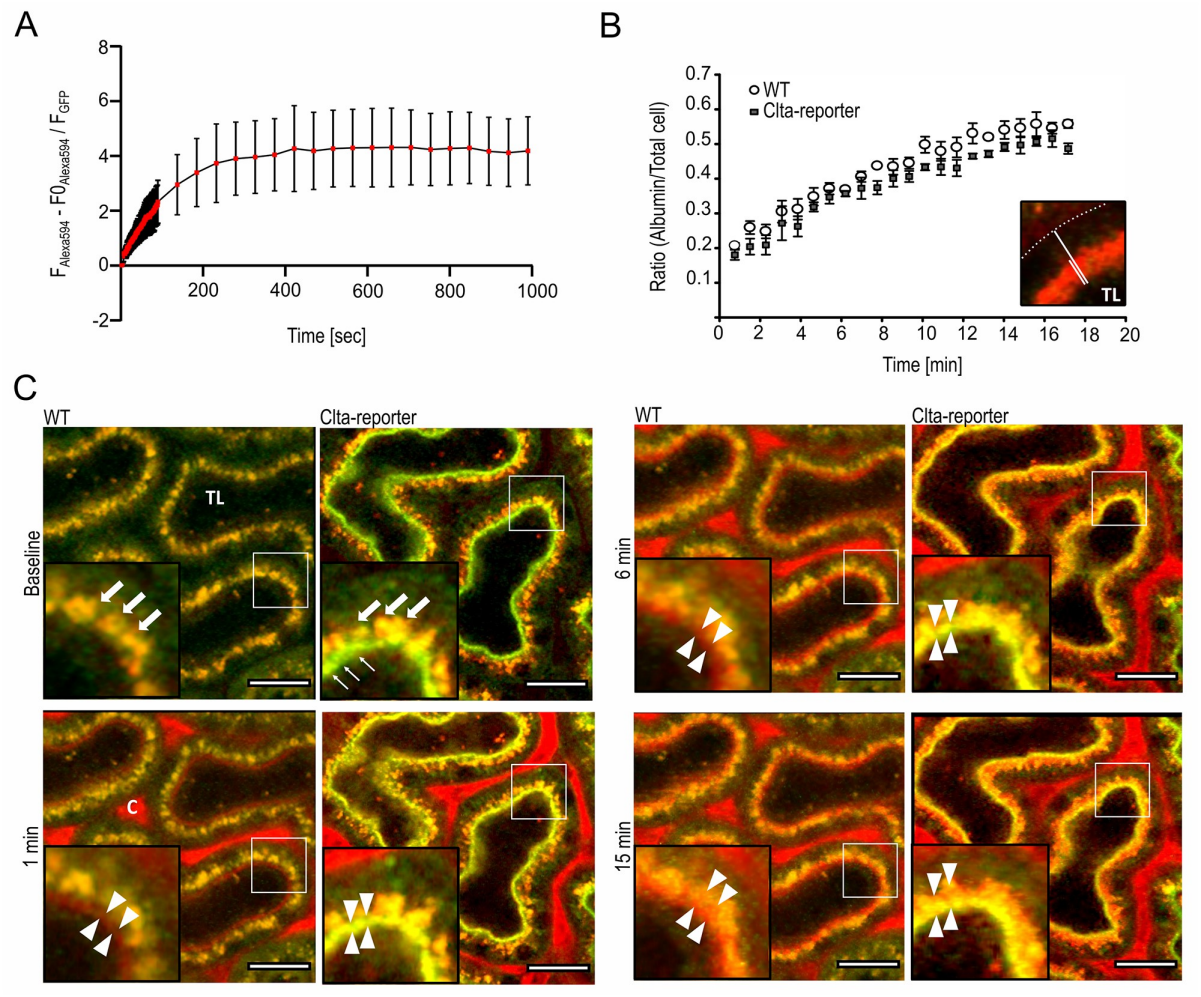
*In vivo* imaging of Alexa594-BSA uptake in Clta-reporter and WT animals. (**A**) Calculated ratio of Alexa594-BSA (background corrected) and Clta-eGFP signal at the apical area in PTEC. Alexa594-BSA quickly accumulates in the Clta-eGFP-positive region of S1 proximal tubules, before a steady state of albumin influx and outflux is reached. A one-phase association non-linear fitting determined a half-time for Alexa594-albumin in the Clta-eGFP-positive proximal tubule compartment of 85.53 seconds. Analysis was performed for Clta-reporter animals only, as the defined eGFP signal at the brush border was used for segmentation of the subapical area. Graph shows mean ± SEM. (**B**) Analysis of Alexa594-BSA uptake in Clta-reporter and WT animals over time was assessed as the ratio of the thickness of Alexa594-albumin-fluorescent region over the total thickness of the respective PTEC for each time point (B inlet, for different time points see S6 Fig in S1 Appendix). No significant difference can be detected at any time point tested (2-way Anova multiple comparison analysis). Dotted line represents basal cell border. TL: lumen. (**C**) Intravital 2-photon microscopy images of WT and Clta-reporter mouse S1 segment proximal tubules (PTs), before (baseline) and 1 min, 6 min and 15 min post i.v. injection of Alexa594-BSA (red). Compared to WT PTs, Clta-reporter mice identify clathrin by bright eGFP-expression (green) in the brush border (baseline, arrows). Both genotypes reveal autofluorescent lysosomes (yellow) in the apical region of PTECs (baseline, bold arrows). 1 min after injection, Alexa594-BSA is clearly visible in peritubular capillaries (c) and accumulates in the brush border of WT (red) and Clta-reporter (yellow) PTs (arrowheads). Over time, proximal tubular uptake and intracellular trafficking of Alexa594-BSA, leads to increased thickness of red fluorescent area in the apical region of PT cells (arrowheads), until Alexa594-BSA is clearly visible in PT lysosomes at 15 min. TL: lumen; Scale bar: 20 μm.

The main rationale in generating this CME-reporter mouse model was the ability to study the dynamics of CME using intravital microscopy. The PTEC of murine kidney are specialized in the re-uptake of proteins and xenobiotics (e.g., Vitamin D binding protein, albumin, or gentamicin) which cross the glomerular filter [43, 44]. CME by megalin and cubilin is an important pathway by which the re-uptake of albumin takes place [38, 45]. This important biological role makes the kidney a promising model to monitor CME *in vivo*. Using intravital two-photon microscopy (2-PM), the uptake of Alexa594-labelled bovine serum albumin (BSA) was monitored in kidneys of Clta-reporter and WT littermates with an implanted abdominal imaging window (AIW). After baseline imaging, Alexa594-BSA was injected i.v. and a time series was recorded (first 90 s at 1frame per second (fps) and then 15 min at 1frame every 47 s) to capture *in vivo* proximal tubular albumin uptake in the living kidney (Fig 6 and S2 and S3 Movies).

We first aimed to address Alexa594-BSA uptake dynamics in the bright Clta-eGFP-positive subapical part of the PTEC membrane. Hereford, the thin eGFP-positive band in S1-PT segments was segmented using Ilastik, and changes of normalized Alexa594-BSA fluorescence determined in the segmented region were assessed over time. After injection of Alexa594-BSA, we first observed a steep accumulation of the dye in Clta-eGFP-segmented area, which then reached a plateau, suggesting steady state of Alexa594-BSA influx and outflux at the subapical Clta-eGFP-positive region of the PTECs (Fig 6A). Alexa594-BSA dynamics fitted a one-phase association non-linear fitting (r2 = 0.5844) and identified a half-life of 85.53 sec. This analysis could be performed in Clta-reporter only, as the defined eGFP signal was used to segment the apical area. In order to assess whether there are physiological changes in Alexa594-BSA uptake and intracellular trafficking in Clta-reporter animals as compared to WT, we next quantified the extent of Alexa594-BSA uptake by determining the thickness of apical Alexa594-BSA fluorescence in S1 PTECs in relation to the overall cell width over time (Fig 6B and S6 Fig in S1 Appendix), as previously described [46]. When comparing the Alexa594-BSA-uptake in Clta-reporter animals and WT littermates, no significant differences between the means of each analysed time point was detected (Fig 6B).

After i.v. injection, Alexa594-BSA quickly appeared in the apical brush border in both genotypes, showing clear co-localization with Clta-eGFP in Clta-reporter kidneys (Fig 6C). Subsequent to albumin uptake and intracellular trafficking, Alexa594-BSA accumulated intracellularly in vesicular structures, leading to an increase of the fluorescent-positive area toward the basal lamina (Fig 6C). These findings demonstrate that the Clta-eGFP fusion protein can be monitored over time and more importantly does reflect physiological CME *in vivo*.

## Discussion

Here we report the generation and characterization of a new reporter mouse model for CME to monitor this process *in vitro* and *in vivo*. The endogenous expression of the Clta-eGFP fusion protein was achieved by using the CRISPR/Cas9 technique. Therefore, the cDNA of eGFP was introduced endogenously in-frame, separated by a flexible linker C-terminally into the murine Clta locus (see Fig 1A). The efficiency, ~30% for KI, was similar to the one reported by Miura *et al*. (Fig 1C). However, there is a higher probability of mutations when using ssDNA as donor [47]. We detected two point-mutations in the eGFP sequence of animal 5. In contrast mosaic animals were not detected. The reason might be the use of the Cas9 protein in the injection mixture [48]. Using Southern Blot analysis, a large deletion downstream of the sgRNA mediated cut site in founder 1 was detected (Fig 1D and S1A Fig in S1 Appendix). This leads to the loss of the last exon of Clta what prompted us to the conclusion that the remaining Clta-eGFP fusion protein is sufficient to fulfill the functions of Clta. Further genetic characterization of this new Clta-eGFP reporter mouse line included analysis of possible off-targets, even though they are described to be rare in CRISPR/Ca9 modified mice [49, 50]. Moreover, off-targets can be out-crossed if not present on the same chromosome as the target region. Using PCR and subsequent sequencing none of the tested off-targets were edited (S1C Fig in S1 Appendix); however large deletions cannot be detected using this approach.

Using western blot analysis of different organs and primary MEFs (Figs 2A and 4A and S3A Fig in S1 Appendix) the Clta-eGFP fusion protein was detected. An additional band of about 40 to 50 kDa was detected in tissue lysates of small intestine and kidney. This additional band could be a result of tissue specific alternative splicing in these organs, but also exon-skipping due to the artificial introduced eGFP would be possible [51]. Partial degradation to a slightly smaller band, as observed in intestine, however, cannot fully be excluded. The endogenous Clta-eGFP fusion protein was further detected in IF of kidney, small intestine (Fig 2B–2D) as well as spleen, heart, lung, and liver (S3B-S3E Fig in S1 Appendix). In highly polarized tissue, that is specialized in (re-)uptake processes, the Clta-eGFP fusion protein shows a strong apical localization (Figs 2B–2D and 6C). In addition, we observed that the expression of Clta-eGFP in collecting duct is either absent or below the detection limit and further investigations are needed (Fig 3). This shows that detecting the endogenous Clta-eGFP fluorescence is more specific than an antibody staining and could be used to specifically investigate the tissue distribution of Clta and thereby tissue-specific roles of Clta, as to our knowledge there is no specific Clta or Cltb antibody available. Liver, spleen, heart and lung showed high autofluorescence in the IF of WT cryo slices and the difference to the Clta-eGFP animals was hardly detectable. Therefore, emission spectra were acquired from the tissue sections, which revealed an emission maximum at 550 nm and a similar curve progression for the autofluorescence in all tested organs in the green spectral range (Fig 2B and S3B-S3E Fig in S1 Appendix). The autofluorescence can be removed by spectral separation algorithms and the remaining signal can be considered as the Clta-eGFP signal. Co-localization analysis, Co-IP and qPCR (S4A Fig in S1 Appendix and Fig 4B and 4C) showed that the eGFP is not interfering with the endogenous Clta function. Although there was no significant difference in the relative expression of Clta and Cltb between WT and Clta-reporter there was a trend to decreased Clta/Cltb ratio.

The pattern of Clta-eGFP in dynamic puncta and their dynamics (Fig 5) are indicative of the correct recruitment of this labelled subunit to clathrin coated pits and vesicles, which bud off the membrane. The subunit also colocalized with nanobeads on the substrate that were previously shown to recruit the clathrin machinery [24] with a lifetime on the beads that is consistent with formation of coated pit formation. This and the finding, that transferrin uptake was the same in wild type and Clta-eGFP MEFs (Fig 4D and S5 Fig in S1 Appendix), demonstrates that Clta-eGFP is a faithful reporter of clathrin-coated pit dynamics.

The primary focus for establishing this reporter mouse model was the possibility to use it in *in vivo* imaging approaches, which would facilitate collection of dynamic data *in vivo*. Most of the research about CME uses 2D cell culture systems with labelled and/or immobilized particles. These studies give insights but might not reflect the *in vivo* state, as the immobilized particles led to abortive CME. We used 2-PM of the kidney in anesthetized mice to track CME *in vivo* using Alexa594-BSA. We dynamically monitored the uptake of Alexa594-BSA over time and did not see a significant difference between the means of each analyzed time point in Clta-reporter and WT animals (Fig 6B). Using Clta-reporter only we detected a steady-state of Alexa594-BSA influx and outflux with a half-time of 85.53 sec (Fig 6A). This parameter might depend on structural features of albumin and the impact of other sources of albumin (e.g. human or mouse) or mouse-models with defective endocytosis could be investigated for possible changes in the half-time [52, 53]. Furthermore, colocalization of Alexa594-BSA and Clta-eGFP fluorescence over time demonstrated CME-uptake and uncoating to mainly be restricted to a narrow band below the apical brush border, whereas below that area Alexa594-BSA appeared mainly independent on Clta-eGFP (Fig 6C).

Nowadays, a drawback of the available *in vivo* imaging approaches is the limitation in either spatial or temporal resolution. For tracking individual CME events a high spatial and temporal resolution is necessary. With our 2-PM approach we were limited to the theoretical lateral resolution of 437 nm (r_xy_ = 0.61λ/NA, where r is the resolution and NA the numerical aperture, here 1.2) [54]. Using STED, the resolution can be enhanced and Clta-eGFP appears in clusters of 74 nm in size or smaller in tissue sections (Fig 2C and 2D). In terms of spatial resolution *in vivo* STED is an alternative [55, 56]. However, the superior spatial resolution is accompanied by a low temporal resolution. To dynamically track single endocytic events *in vivo* spatial and at the same time temporal resolution might be important, demanding instrumental development, and extension of lattice-light sheet with adaptive optics to mammalian systems could be an appropriate approach to follow [57]. Nevertheless, there is a huge number of examples in the literature where these mice can already help to answer questions in basic (e.g. the role of clathrin plaques in vivo [58]) and disease-associated (e.g. viral entry of HIV [59, 60], SARS-CoV [61], SARS-CoV2 [62, 63]; impact on acute kidney injury (AKI) [64]) research about CME. Tubular damage and progression of renal diseases are associated with a massive amount of proteins that have to be reabsorbed in PTEC. The reabsorption mainly occurs via receptor-mediated endocytosis and the contribution of CME to AKI can be investigated with these mice [64]. Furthermore, the Clta-reporter can be also used to investigate the bioavailability of CME inhibitors (e.g. in schizophrenia and bipolar disorders [65]) or to monitor drug delivery via CME of nanoparticle delivered drugs [66]. In addition to that the Clta-reporter mice can be crossed with other (disease-associated) mouse strains and investigate the impact of endocytosis in disease progression (e.g. schizophrenia) [65, 67]. Furthermore, our finding of specific Clta-eGFP expression in proximal tubules but not collecting duct of the kidney (Fig 3) hint to an isoform specific role of Clta, which attracted more and more attention over the last few years and which could be studied with the help of these mice [21–23, 68, 69].

All in all, we are convinced, the Clta-reporter mouse model is a suitable and powerful tool (currently with some technical limitations) to dynamically track endocytic processes *in vivo*.

## Material and methods

### Animals

All animal experiments were approved by the local regulating authorities (Regierungspräsidium Karlsruhe and the Danish Ministry of Justice (Dyreforsøgstilsynet) and by internal DKFZ committees. The mice were kept in specific-pathogen-free facilities.

### Cell culture

HeLa cells were grown at 37°C and 5% CO_2_ in a humidified incubator in Dulbecco’s modified eagle’s medium (DMEM) supplemented with 10% of fetal calf serum (FCS) and 1% of penicillin-streptomycin (Pen/Strep). For transfection, cells were seeded in 12-well plates at a confluency of 50%.

Mouse embryonic fibroblasts (MEF) were grown at 37°C and 5% CO_2_ in a humidified incubator in DMEM supplemented with 10% of FCS, 1% of Pen/Strep and 1% of 1 M HEPES (pH 7.4). For Western Blot and Co-Immunoprecipitation cells were seeded at a confluency of 60% and cultured until confluency was reached. For Immunofluorescence cells were seeded, fixed, and stained directly on glass coverslips.

### CRISPR/Cas9-mediated β-Gal gene complementation assay

Single guide RNAs (sgRNAs) were designed to target the stop codon of murine Clta using CRISPOR.tefor.net web tool [34]. The sgRNAs (sgRNA D1: GGCTCTTCAGTGCACCAGGG; sgRNA D2: GACGTAGTGTTTCCACAGGG; and sgRNA D3: GTAGACGTAGTGTTTCCACA) were cloned in the pX330-U6-Chimeric_BB-CBh-hSpCas9 (a gift from Feng Zhang, Addgene plasmid #42230) [6]. The genomic region where the sgRNAs cut (5’AGCAGGCCCCCCTGGTGCACTGAAGAGCCACCCTGTGGAAACACTACGTCTACAAT3’) was introduced into pCMV-TALEN-Rep (a gift from Ralf Kuehn, Addgene plasmid #45964). HeLa cells were transfected with pCMV-TALEN-Rep, pX330-sgRNAD1-3 and a luciferase reporter plasmid (Pierce Cypridina Lucifersae Glow Assay Kit) using Metafectene ^®^Pro (Biontex). After cell lysis the β-Gal gene complementation assay was started by the addition of ortho-Nitrophenyl-β-galactopyranoside (OPNG) and incubated at 37°C while shaking. The reaction was stopped by the addition of Na_2_CO_3_ and enzyme activity was measured at 420 nm. The expression of Cypridina luciferase was measured according to manufacturer’s protocol and used for normalization.

### Generation of the Clta-reporter animals

The sgRNA was ordered as Alt-R^®^ CRISPR-Cas9 crRNA (5’GTAGACGTAGTGTTTCCACA3’, resulting sequence: /AltR1/rGrU rArGrA rCrGrU rArGrU rGrUrU rUrCrC rArCrA rGrUrU rUrUrA rGrArG rCrUrA rUrGrC rU/AltR2/) and Alt-R^®^ CRISPR-Cas9 tracrRNA from IDT. The gRNA was prepared by annealing crRNA and tracrRNA as described by Miura *et al*. [8]. The ssDNA donor (S1 File) was ordered from GeneWiz. The crRNA-tracrRNA complex, ssDNA donor and the Alt-R^®^ S.p. Cas9 Nuclease V3 (IDT) were diluted in microinjection buffer (100 μL) and 2 pL were injected pronuclear into zygotes and implanted into foster mothers. For details of the injection mixture see Table 1.

**Table 1:** Injection mixture for pronuclear injection.

compound	Final concentration
crRNA-tracRNA	10+10 ng/μL
Cas9 protein	20 ng/μL
ssDNA donor	10 ng/μL

### Genotyping

The DNA of an ear punch of the animals was isolated. Afterwards correct integration was confirmed using the following primers binding upstream and downstream of the inserted region Clta-KI-for 5’CAA ACC TGT ACT CCG CCA TT3’ and Clta-KI-rev 5’-CTC TGA ATG CCA GGG AGA AC-3‘ using the Q5^®^-Polymerase (New England Biolabs) and the following cycling conditions 98°C for 1 min; 98°C for 20 sec, 66°C for 20sec and 72°C for 1 min 30 sec (35x) and 72°C for 5 min. For detection of the deletion in founder 1 and half of its offsprings Clta-KI-rev and Clta-upper-for 5‘-GCA GCA CAA TAT TAC CAG AGT C-3‘ were used.

### Immunofluorescence

Clta-reporter animals were anesthetized using a mixture of Ketamin (120mg/kg body weight) and Xylazin (16mg/kg body weight) and transcardially perfused with 4% formalin in 1x PBS. The organs were incubated overnight in 4% formalin. For cryoprotection, organs were incubated successively in 15% sucrose and 30% sucrose overnight, frozen in Tissue-Tek^®^ O.C.T.^™^ Compound (Sakura Finetek) and stored at -80°C. Cryosections were fixed in 4% formalin (in 1x PBS) at RT for 5 min. The organ sections were incubated with the following primary antibodies AQP1 (sc-20810; 1:25), AQP2 (sc-9882, 1:800) and megalin (sc-16478, 1:25) at 37°C for 1h, followed by a 30 min incubation at 37°C with Alexa Fluor^™^ 633 donkey anti-goat (1:200), Alexa Fluor^™^ 633 goat anti-rabbit (1:200) or Alexa Fluor^™^ 555 goat anti-rabbit (1:200). For visualization of the nuclei, tissue sections were stained with DAPI.

Multiphoton Microscopy (Two-Photon-Microscopy, 2PM) was performed on a Zeiss LSM 780 NLO equipped with a Mai Tai HPDS femtosecond laser (Spectra Physics). The specimens were excited at 900nm and scans were recorded by a 63x/1.40 oil immersion objective lens and a 32-channel GaAsP spectral detector in the range of 419-647nm. To remove the strong autofluorescence background signal that masked the eGFP fluorescence from the final image, a spectral unmixing algorithm was used. This method determines the spectral overlap of fluorochrome emission signals and eliminates crosstalk between adjacent channels. The prerequisite for the spectral unmixing procedure is the knowledge of both reference spectra: The reference spectrum for eGFP was taken from the built-in dye database. The autofluorescence emission spectrum was obtained from a tissue sample of the WT littermate that does not contain any eGFP signal by the spectral detector. The Clta-reporter sample with the mixed signal was then imaged accordingly with the same recording parameters. The Spectral Unmixing plug-in within the Zeiss ZEN software was finally used to separate both fluorescence signals (eGFP and autofluorescence background).

For co-localization analysis Clta-reporter MEFs and WT MEFs seeded onto glass coverslips were fixed with 4% formalin in 1x PBS for 15 min at RT, blocked in 1% BSA in PBS-T, permeabilized with 0.25% Triton-X-100 in 1x PBS and incubated with the following antibodies mouse anti-CHC (ab2731, 1:100 in 1%BSA in PBS-T) and goat anti-Lc (sc-32518, 1:50 in 1%BSA in PBS-T) or rabbit anti-eGFP (GTX26556, 1:250 in 1%BSA in PBS-T) at 4°C overnight. Cells were incubated with Alexa Fluor^™^ 633 goat anti-mouse (1:200), Alexa Fluor^™^ 488 goat anti-rabbit (1:200) and Alexa Fluor^™^ 546 donkey anti-goat (1:200) secondary antibodies for 2h at RT. To visualize the nuclei, the cells were stained with DAPI. Images were acquired with a Leica TCS SP5 confocal microscope (Leica Biosystems), and subsequent co-localization analysis was performed with the open-source FIJI using the freely available Coloc2 plugin [70].

For the spectral dye separation approach, the organs were removed and incubated in 4% formalin in 1x PBS overnight. For cryoprotection, the tissue was incubated successively in 15% sucrose (1x PBS) and 30% sucrose (1x PBS) overnight, frozen in Tissue-Tek^®^ O.C.T.^™^ Compound (Sakura Finetek) and stored at -80°C. Cryosections were fixed in 4% formalin (1x PBS) at RT for 5 min and stained with DAPI to visualize the nuclei.

Single-photon confocal imaging and dye separation were carried out similarly to the unmixing method described above using a Leica TCS SP5 confocal microscope (Leica Biosystems) and the integrated Spectral Dye Separation plug-in. For the unmixing procedure, the respective reference spectra must be known (as for eGFP) or determined by a so-called Lambda Scan, in which the emission curve is recorded by means of a narrow detection window that is being spectrally shifted (as for autofluorescence). Just as in the 2PM experiment, the reference spectrum for eGFP was taken from the built-in dye database and the autofluorescence emission spectrum was recorded from a tissue sample of the WT littermate that does not contain any eGFP signal: The specimen was excited with an argon laser line of 475 nm, and the emission was successively registered in 5 nm steps with a 10 nm wide detection window in the detection range of 490–750 nm. The same acquisition parameters were applied to the Clta-reporter sample containing the mixed signal. Finally, the two reference spectra were used to generate two unmixed channels (eGFP and autofluorescence background) using the same plug-in.

### Isolation of primary mouse embryonic fibroblasts

Clta-reporter animals were crossed with C57BL/6N animals. Pregnant female animals were euthanized by cervical dislocation at E14.5. The embryos were harvested by decapitation, washed and all blood-carrying tissue was removed. Embryos were digested using 2.5% Trypsin (ThermoFisher Scientific) containing 5 mM EDTA at 37°C. The digestion was stopped by the addition of DMEM containing 10% FCS, 1%Pen/Strep and 1% HEPES. The cell suspension was suspended with cannulas of different sizes (18G x 1 ½”, 20G x 1 ½” and 22G x 1 ½”), filtered through a 70 μm cell strainer and cultured at 37°C and 5% CO_2_ in an humified incubator. For genotyping of the individual primary MEF cell lines see section Genotyping.

### Immunoprecipitation

MEFs from a 15 cm confluent petri dish were harvested with a rubber policeman, centrifuged, frozen in liquid nitrogen and lysed in IP cell lysis buffer (50 mM Hepes (pH 7.5), 50 mM NaCl, 1.5 mM MgCl2, 5 mM EGTA, 1% Triton X-100, and 5% glycerol) containing 1x Complete Protease Inhibitor (Roche). Protein concentration was adjusted to equal amounts and lysis buffer diluted 1:2 with IP-wash buffer (50 mM Hepes (pH 7.5), 50 mM NaCl, 1.5 mM MgCl2, 5 mM EGTA, 0.05% Triton X-100). 25 μl of GFP-Trap Magnetic Agarose (Chromotek, gtma-10) were added to Clta-reporter and WT MEF lysates and incubated in an overhead shaker at 4°C for 2h. Beads were separated from liquid with a magnet followed by three washing steps with IP-wash buffer. Immuno-absorbed proteins were eluted from beads in SDS-sample buffer at 95°C and identified using SDS-PAGE and subsequent Western Blot. Primary antibodies used were rabbit anti-CHC (Cell Signaling, 1:1000 in 5% BSA in TBS-T); rabbit anti-eGFP (GTX26556, 1:5000 in 5% BSA in TBS-T) and rabbit anti-beta-Tubulin (Cell Signaling, 1:2000 in 5% BSA in TBS-T) followed by subsequent incubation with an HRP-conjugated secondary goat anti-rabbit antibody (1:1000 in 5% BSA in TBS-T).

### Western Blot

Tissue and cells were frozen in liquid nitrogen and homogenized in cell lysis buffer (20 mM HEPES-NaOH (pH 7.4), 25 mM KCl, 250 mM sucrose, 2 mM MgCl_2_, 0.5 mM DTT and 1% digitonin (Sigma)) containing 1x Complete Protease Inhibitor and 1x PhosStop (Roche) using the Qiagen TissueLyser II. Tissue lysates were cleared by centrifugation, separated by SDS-PAGE, and transferred to nitrocellulose membranes. Membranes were blocked with 5% skim milk or 5% BSA in 1xTBS containing 0.1% Tween-20 for 1h at RT and incubated overnight with one of the following primary antibodies: rabbit anti-eGFP (GTX26556, 1:5000 in 5% milk in TBS-T), mouse anti-Lc (MA5-11860, 1:1000 in 5% milk in TBS-T), mouse-anti-GAPDH (sc-25778, 1:1000 in 5% milk in TBS-T), rabbit anti-CHC (Cell Signaling, 1:1000 in 5% BSA in TBS-T) or beta-Tubulin (Cell Signaling, 1:2000 in 5% BSA in TBS-T) followed by subsequent incubation with an HRP-conjugated secondary goat anti-rabbit or goat anti-mouse antibody (1:3000 in 5% milk or 5% BSA in TBS-T). Membranes were visualized using Clarity^™^ Western ECL substrate (Biorad) and ChemiDoc^™^ MP Imaging System (Biorad).

### Southern Blot

The correct integration was validated using Southern Blot. The Sothern Blot probe was synthesized according to manufacturer’s protocol using the PCR DIG probe synthesis kit (Roche) using the following primers: Clta-NdeI-for 5’-GTA TCA GCC AAG CCA TTG GT-3‘ and Clta-NdeI-rev: 5‘-TGG GCA TGG AAA AAG AAG TC-3‘. DNA from animal tissue was isolated, digested with *Sca*I at 37°C overnight, following the recommendations of the manufacturer, separated by agarose gel electrophoresis, transferred to a positively charged nylon membrane, and finally detected using the Dig luminescent Detection Kit for Nucleic Acids (Roche) and an x-ray film developer machine.

### qPCR

Total RNA from MEFs was isolated using the RNeasy^®^ Micro Kit (Qiagen) following ma-nufacturer’s protocol. Single-stranded cDNA was synthesized using the SuperScript^™^ double-stranded cDNA synthesis kit (Invitrogen) following manufacturer’s protocol. RT-qPCR was performed using LightCycler-FastStart DNA MasterSYBR Green I kit (Roche) and LightCycler^®^ (System 2.0, Roche) following manufacturer’s protocol. The relative expression of Clta and Cltb was determined using the following primers mClta-for 5’-GGA AGC CCT CGA TGC CAA T-3’, mClta-rev 5’-ACA AAG GCT TCT TCT GCT GC-3’, mCltb-for 5’-GAT CAA CAA CAG GGC ATC GG-3‘, mCltb-rev 5’-GCA GGC GGG ACA CAT CTT TA-3’, mGapdh-for 5’-ACT CCC ACT CTT CCA CCT TC-3’, mGapdh-rev 5’-GGT CCA GGG TTT CTT ACT CC-3’, m18S-for 5’-TGC CCT ATC AAC TTT CGA TGG TA-3’, and m18S-rev 5’-CAA TTA CAG GGC CTC CAA AGA GT-3’ and the comparative CT method, normalizing gene expression to the housekeeping genes mGapdh and m18S.

### Surface immobilization of beads

For covalent immobilization of 200 nm-sized beads, glass coverslips were modified by chemical functionalization. QGel920 elastomers (CHT Silicones, Richmond VA) were prepared as previously described [71]. Briefly, QGel920 Part A and QGel920 Part B were mixed in the ratio 1:1 (corresponding to around 3 kPa) and coated onto a 24 mm coverslip using a spin-coating machine (5000 rpm; Laurell Technologies Corporation). Each coverslip was then baked at 70 degrees overnight. For functionalization, the elastomer-coated coverslips were treated for 5 min with 3-aminopropyl trimethoxysilane (APTES, 10% v/v) and washed once with ethanol and twice with water. To covalently link the beads to the substrate, 200 nm carboxylate-modified dark red FluoSpheresTM (Thermo Fisher Scientific) were mixed with 0.1 mg/ml 1-Ethyl-3-(3-dimethylaminopropyl) carbodiimide (EDC) in water and incubated on the substrate for 1h at room temperature. Afterwards, surfaces were rinsed twice with sterile PBS before cell seeding.

### TIRF-microscopy of Clta-eGFP recruitment to coated beads

MEFs were trypsinized and replated on glass coverslips with beads and allowed to settle for 30 minutes before imaging on a Nikon Ti2 inverted microscope equipped with H-TIRF illumination using Nis-Elements 5.2 (Nikon Instruments, Europe). Clta-eGFP was excited using the 488 nm and beads with the 647 nm laser, respectively. Emission was captured on an EM-CCD (Andor iXon 897) at approx. 1 second intervals for several minutes. Cells were imaged 30 minutes to 2 hours after plating.

For analysis the GFP signal was denoised using the denoise AI function of Nis-Elements and sequences were corrected for stage drift using the beads as fiduciaries. For quantification of Clta-eGFP recruitment on beads, a mask was placed over the beads in the 640 channel (0.41 μm^2^) and average intensity plotted over time. Lifetime of Clta-GFP on beads was measured in 6 cells, for 25 beads. Values clustered into two classes and therefore grouped accordingly (lifetimes either shorter or longer than 110 seconds).

### Cy3Tf uptake assay

Clta-reporter and WT MEFs were seeded in 96-well plates at a density of 20,000 cells/well and cultured overnight at 37°C and 5% CO_2_ in a humidified incubator. After washing with PBS cells were incubated with 20 μg/mL of Cy3Tf (015-160-050, Jackson-Immunoresearch) for 30 min, followed by an acid wash (pH 2.5, 150 mM NaCl, 1 mM MgCl_2_, 0.125 mM CaCl_2_ and 100 mM glycine) to remove surface-bound Cy3Tf. After lysis (20 mM HEPES-NaOH (pH 7.4), 25 mM KCl, 250 mM sucrose, 2 mM MgCl_2_, 0.5 mM DTT and 1% triton-X-100) containing 1x Complete Protease Inhibitor and 1x PhosStop (Roche), fluorescence readout was performed using a Synergy H1 microplate reader (BioTek).

### Inhibition of Cy3Tf uptake

Clta-reporter and WT MEFs were seeded onto glass coverslips. The cells were pre-treated with 5 μg/mL of chlorpromazine (CPZ) for 15 min to inhibit Cy3Tf uptake. The Cy3Tf uptake assay was performed as described above in the presence of 5 μg/mL CPZ. After fixation and Dapi staining Cy3-Tf was visualized using a Leica TCS SP5 confocal microscope.

### STED microscopy

The tissue sections of Clta-reporter and WT animals were prepared as already described in the immunofluorescence section. STED microscopy was performed on a home-built setup [72] with the following settings: eGFP was excited at 483 nm with an average power of 12 μW and depleted at 595 nm with 21 mW average power at the back aperture of the objective lens (PL APO, 1.3 numerical aperture, glycerol immersion; Leica, Wetzlar, Germany). STED images were recorded with 30 × 30 nm pixel size and 20 μs pixel dwell-time. STED and corresponding confocal images were smoothed with a low-pass gauss filter in the acquisition software Imspector (Abberior Instruments). Images of Clta-reporter and WT mice were recorded with the same settings.

### *In vivo* imaging 2PM and analysis

12 weeks old Clta-reporter mice and WT littermates (n = 3, each) received buprenorphine [0.1 mg/kg BW, s.c.] prior to surgery on an operating table with a servo-controlled heating plate under isoflurane-mediated anesthesia [1 vol. %]. To facilitate imaging of the kidney, an abdominal imaging window (AIW) was implanted as described before [73, 74]. Briefly, a 1 cm long dorsoventral incision was placed above the left kidney, which is then surrounded by a purse-string suture attaching the skin to the muscle layer of the abdominal wall. The kidney was gently mobilized and then glued to the glass front of a sterile, cover-slipped titanium ring, which was inserted into the abdominal wall and fixed by closure of the purse-string suture. A tail vein catheter was inserted for i.v. administration of Alexa594-BSA (ThermoFisher). After surgical intervention, the anesthetized animals were directly transferred on a Bruker Ultima Investigator two photon microscope equipped with a heating pad to maintain physiological body temperature. Continuous isoflurane-mediated anesthesia was maintained during the subsequent imaging session. Visualization of the kidney was facilitated through the implanted AIW using an Olympus UPLSAPO60XW 60x/1.1 W.D = 0.28mm objective and exciting at 860 nm with a Chameleon Ultra-II multiphoton laser (Coherent). Fluorescent emission was collected using GaAsP non-descanned detectors with the following spectral detection windows 570–620 nm for Alexa594-BSA, 500–550 nm for Clta-eGFP and 435–485 nm for kidney autofluorescence. After locating of a well-suited field of view, baseline imaging was performed and subsequently Alexa594-BSA was injected to investigate S1 proximal tubule albumin uptake dynamics. Hereford, a t-series was recorded in two phases. The first 90s with 1 frame per second and then 15 min with 1 frame every 47 s. Subsequent to image acquisition, the anesthetized animals were perfusion-fixated for tissue collection.

Image processing and analysis were performed by the freely available open-source FIJI [70]. First, image denoising was performed using PureDenoise and second, image stabilization was performed to compensate for sample drift using the descriptor-based series registration [75, 76].

Alexa594-BSA uptake and colocalization with Clta-eGFP at the brush border of the proximal tubule in Clta-reporter mice was quantified with a ratiometric approach. The Clta-eGFP-positive area was segmented in Ilastik [77] in a two-stage process. Imaging data was duplicated and normalized with a Z-Score approach by subtracting the mean intensity and dividing by the standard deviation of the mean for each frame in order. This was done to enhance the segmentation accuracy by compensating for changes in intensity over time resulting from Alexa594-BSA uptake and accumulation in the PTECs. During the first segmentation step, PTECs were isolated from vasculature and tubular lumen, in the second step, the Clta-eGFP-positive region was segmented from the PTECs cytoplasm. The segmentation produced stencils that were applied on the data in order to selectively quantify raw Alexa594-BSA and Clta-eGFP fluorescence intensities in the segmented area. To compensate for slight focal plane changes induced by breathing movements which generated oscillations in the Alexa594-BSA signal, Clta-eGFP was used a reference signal to calculate a Alexa594-BSA/Clta-eGFP ratio over time. For each experimental subject, 2 proximal S1 tubules for a total of 6 segments were quantified and then nonlinearly fitted in Graphpad Prism 9 using a one phase association model.

To determine differences of albumin uptake and intracellular trafficking dynamics of WT and Clta-reporter animals, we further assessed the ratio between the length of apically labeled Alexa594-BSA region and the entire cellular cross-section length of respective PTECs in a selected S1 segment as previously described [46].

## Supporting information

S1 Appendix Supporting information. This supporting information includes figures S1 to S6 and their figure legends as well as the legends to the supporting movies S1 to S3.
(PDF)

S1 File Sequence of the single stranded DNA donor (ssDNA donor). (PDF)

S1 Movie TIRFM of Clta-eGFP in the cell shown in Fig 5A–5C. (AVI)

S2 Movie Time series of Alexa594-BSA uptake in WT kidney. (M4V)

S3 Movie Time series of Alexa594-BSA uptake in Clta-reporter kidney. (M4V)
