# Supplementary material for: A Clathrin light chain A reporter mouse for in vivo imaging of endocytosis
Source: PLoS One. 2022 Sep 23;17(9):e0273660. doi: 10.1371/journal.pone.0273660 (PMC9506643; doi:10.1371/journal.pone.0273660)
Supplement: S1 Appendix — This supporting information includes figures S1 to S6 and their figure legends as well as the legends to the supporting movies S1 to S3. (PDF) [file pone.0273660.s001.pdf]

Supporting information

S1 Figure

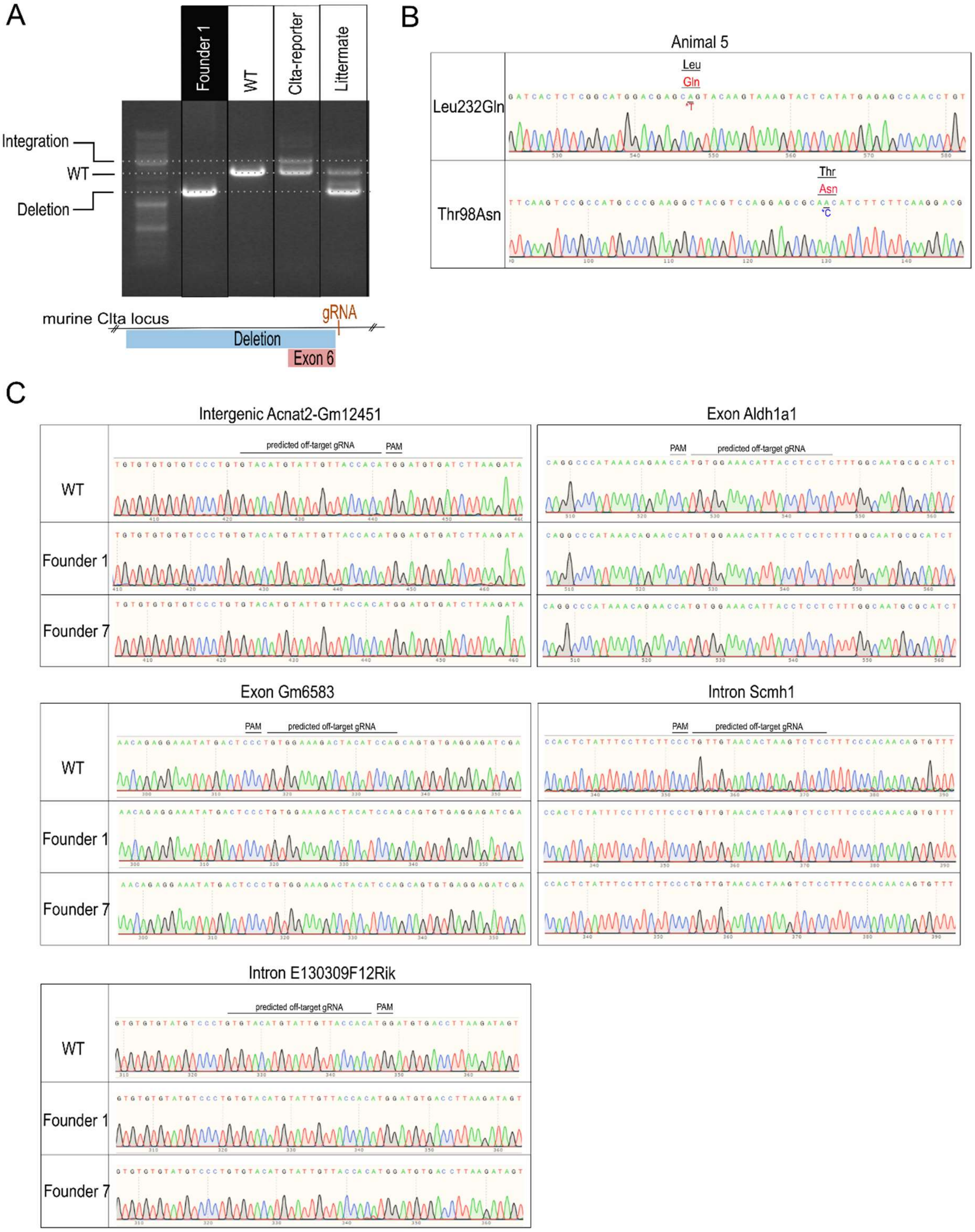

S1 Fig. Genetic validation of the founder lines 1 and 7. (**A**) PCR detecting the deletion on the

second allele in founder 1. An 855 bp deletion, downstream of the sgRNA-mediated cut site was detected. To detect the deletion a different forward primer (see Material and Methods section) was used. Integration band in founder 1 is hardly visible. (**B**) Sequencing confirmed 2 point mutations resulting in amino acid exchanges (Thr98Asn and Leu232Gln) in the eGFP sequence of Animal 5. (**C**) Off-target analysis in founder 1 and founder 7, only some predicted off-targets by CRISPOR.tefor.net were tested. PCR and subsequent sequencing revealed that none of the tested off-targets were edited (large deletions will not be detected with that approach).

S2 Figure

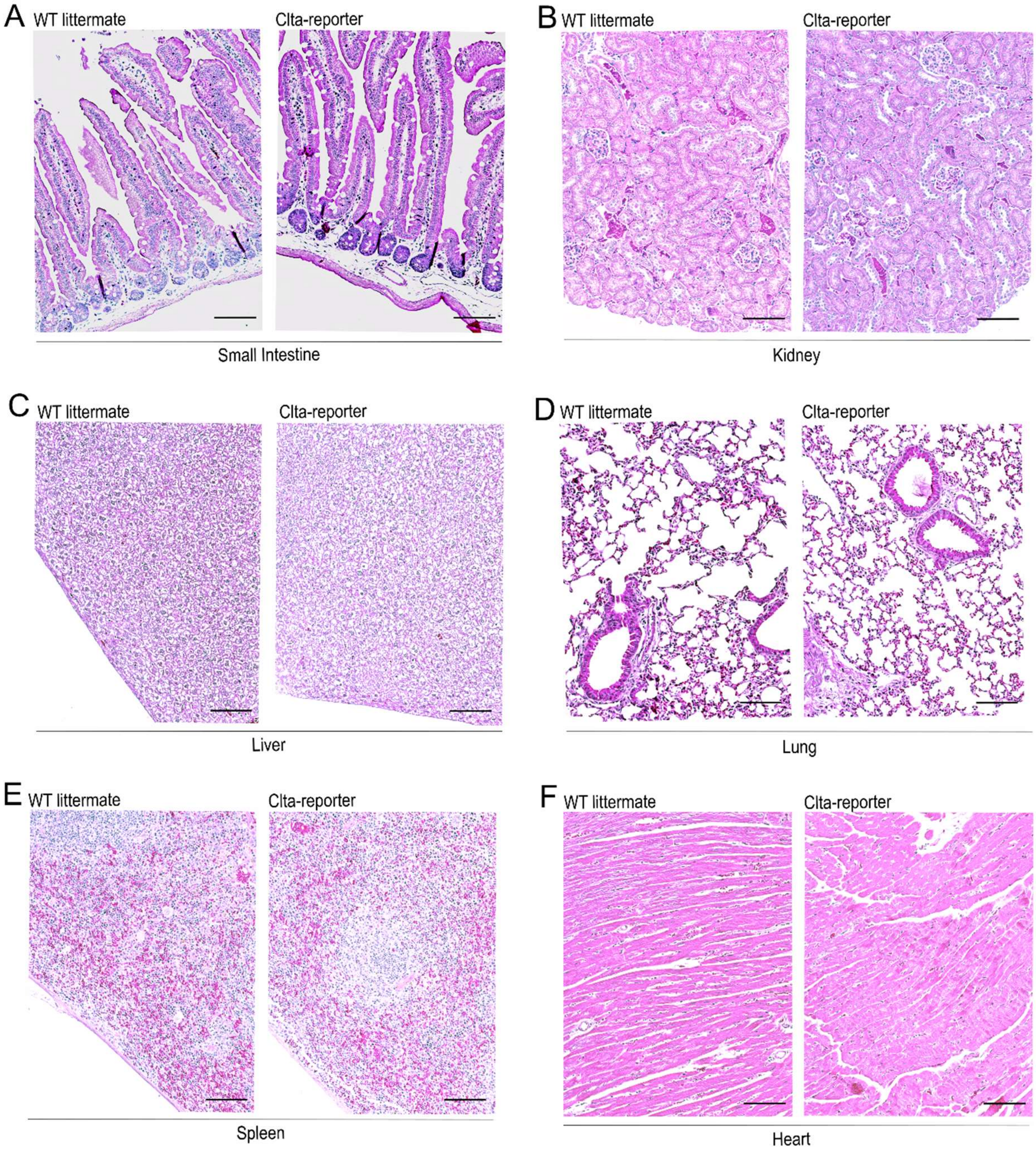

S2 Fig. Histological sections of Clta-reporter and WT. Hematoxylin and Eosin (H&E)-staining of small intestine (**A**), kidney (**B**), liver (**C**), lung (**D**), spleen (**E**) and heart (**F**) did not reveal aberrant morphological alterations. Scale bar: 100 µm.

S3 Figure

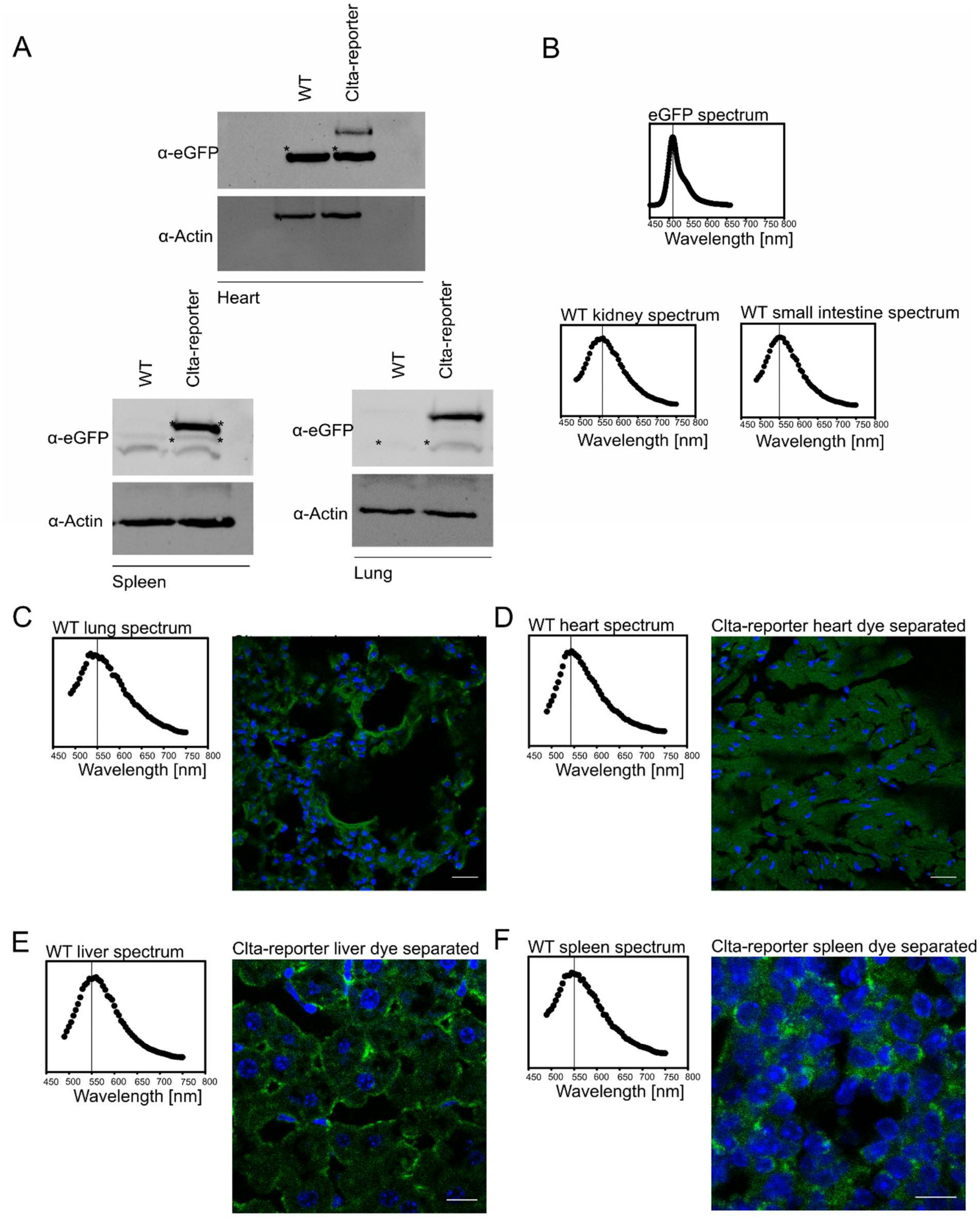

S3 Fig. Expression of the Clta-eGFP fusion protein. (**A**) Expression of the Clta-eGFP fusion protein in heart, lung and spleen is demonstrated on Western Blot. *: unspecific cross reactivity

of the eGFP antibody. (**B**) Emission spectrum of eGFP and WT emission spectra in the green spectral range of kidney (**left**) and small intestine (**right**). (**C-F**) WT emission spectrum in the green spectral range of lung; scale bar: 25 µm (**C**), heart; scale bar: 25 µm (**D**), liver; scale bar: 15 µm (**E**) and spleen; scale bar: 10 µm (**F**), together with the resulting dye separated image of the Clta-eGFP reporter.

S4 Figure

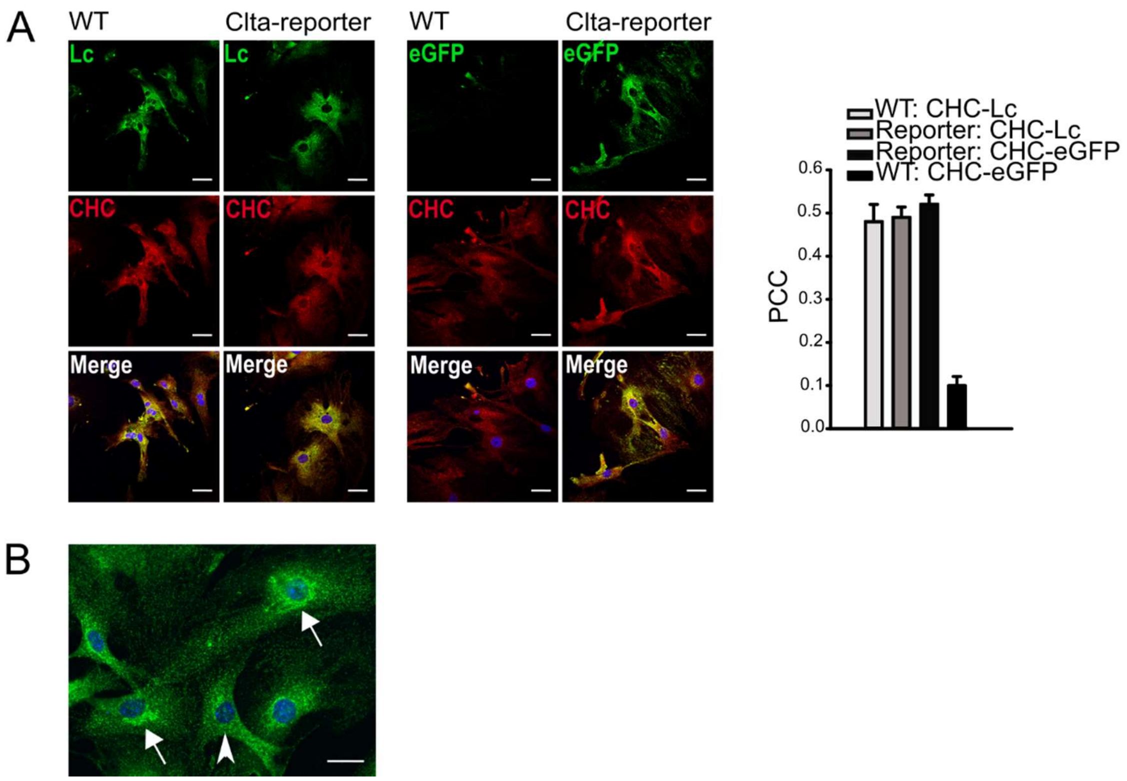

S4 Fig. A) Co-localization of CHC-Lc and CHC-Clta-eGFP double stainings in MEFs (**left**) and subsequent co-localization analysis by calculating Pearson's correlation coefficient (**right**, students t-test). Scale bar: 50 µm. **B)** Primary Clta-eGFP MEFs stained with anti-GFP antibody and visualized with an epifluorescence microscope (Keyence 9000). Note some MEFs show prominent perinuclear staining (arrow) whereas others do not (arrow head). Scale bar: 50 µm.

S5 Figure

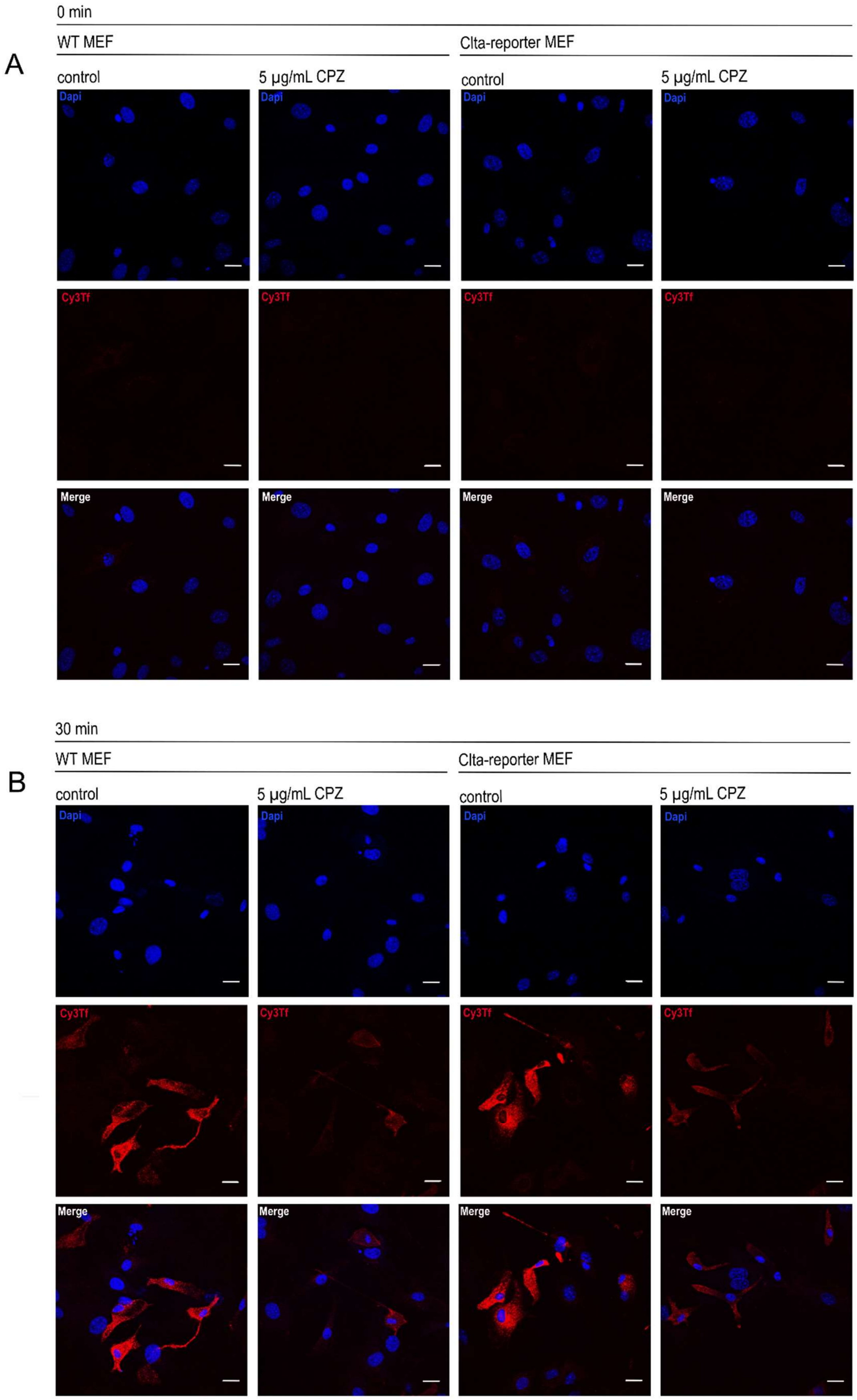
0 min
WT MEF
Clta-reporter MEF
A
control
5 μg/mL CPZ
control
5 μg/mL CPZ
Dapi
Cy3Tf
Merge
30 min
WT MEF
Clta-reporter MEF
B
control
5 μg/mL CPZ
control
5 μg/mL CPZ
Dapi
Cy3Tf
Merge

S5 Fig. Cy3-Tf uptake in CPZ treated MEFs. WT and Clta-reporter MEF were treated 15 min prior and during the Cy3-Tf uptake with 5 µg/mL of CPZ and compared with corresponding control-treated MEF (control). Confocal pictures at 0 min (**A**) and 30 min (**B**) after adding Cy3Tf are shown. Scale bar: 25 µm.

S6 Figure

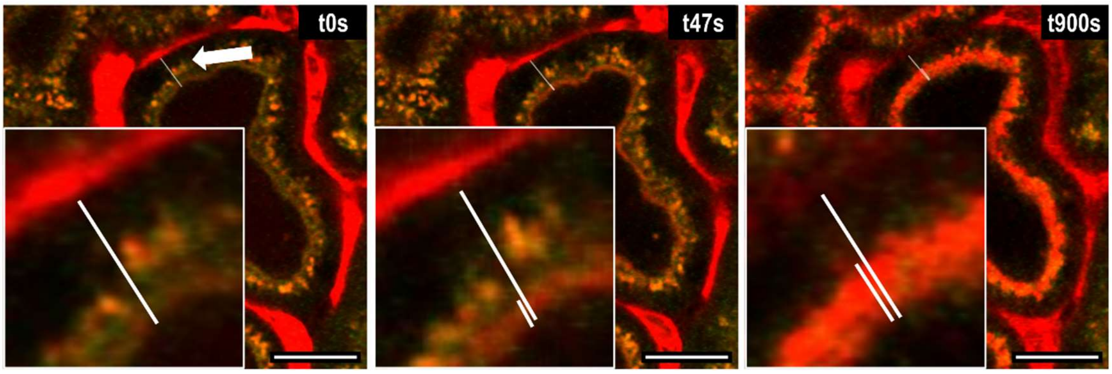

S6 Fig. Determination of Alexa594-BSA uptake and intracellular trafficking dynamics. Ratiometric measurement between the apically labelled Alexa594-BSA region and the entire cellular cross-section length. It is possible to observe the progressive Alexa594-BSA uptake over time. Scale bar: 20 µm.

S1 Movie. TIRFM of Clta-eGFP in the cell shown in Fig. 5A-C. The cell was imaged at approx. 1 pfs. Clta-eGFP puncta show dynamic behaviour. In the boxed region at t=110 a circle highlights the appearance of a Clta-eGFP spot at t=113 seconds and persists over many frames before It gets weaker at t=135 seconds for 2 frames before it disappears.

S2 Movie. Time series of Alexa594-BSA uptake in WT kidney. Accumulation of Alexa594-BSA in the brush border of proximal tubule of murine kidney over time. Scale bar: 25 µm.

S3 Movie. Time series of Alexa594-BSA uptake in Clta-reporter kidney. Accumulation of Alexa594-BSA in the brush border of proximal tubule of murine kidney over time. Co-localization of Clta-eGFP and Alexa594-BSA in Clta-eGFP reporter animals results in the appearance of yellow color at the brush border. At late time points additional red fluorescence is observed a few micrometers below the brush border, which does not overlay with the Clta-eGFP reporter thus demonstrating endocytosed Alexa594-BSA, likely after uncoating of the clathrin cage. Scale bar: 25 µm.

S1 File. Sequence of ssDNA donor. Black: homology arms, purple: additional *Nde*I and ScaI restriction sites, green: eGFP, red: GGSGSVWV-linker. The C-terminal coding sequence of the Clta exon 6 is underlined.
