# Supplementary material for: A Clathrin light chain A reporter mouse for in vivo imaging of endocytosis
Source: PLoS One. 2022 Sep 23;17(9):e0273660. doi: 10.1371/journal.pone.0273660 (PMC9506643; doi:10.1371/journal.pone.0273660)
Supplement: S1 File — (PDF) [file pone.0273660.s002.pdf]

5’CTAAAGACAAAAGGTTTGGTCCAAAAGAACTCAACATAATTAATCCAATTACGTGAACAGCTTCACTGAGTA
GGATTAAGATATTGTAGACGTAGTGTTTCCACAGGtTGGCTCTcatatgagtactTTACTTGTACAGCTCGTCCATGC
CGAGAGTGATCCCGGCGGCGGTCACGAACTCCAGCAGGACCATGTGATCGCGCTTCTCGTTGGGGTCTTTGCT
CAGGGCGGACTGGGTGCTCAGGTAGTGGTTGTCGGGCAGCAGCACGGGGCCGTCGCCGATGGGGGTGTTCT
GCTGGTAGTGGTCGGCGAGCTGCACGCTGCCGTCCTCGATGTTGTGGCGGATCTTGAAGTTCACCTTGATGCC
GTTCTTCTGCTTGTCGGCCATGATATAGACGTTGTGGCTGTTGTAGTTGTACTCCAGCTTGTGCCCCAGGATGTT
GCCGTCCTCCTTGAAGTCGATGCCCTTCAGCTCGATGCGGTTCACCAGGGTGTCGCCCTCGAACTTCACCTCGG
CGCGGGTCTTGTAGTTGCCGTCGTCCTTGAAGAAGATGGTGCGCTCCTGGACGTAGCCTTCGGGCATGGCGGA
CTTGAAGAAGTCGTGCTGCTTCATGTGGTCGGGGTAGCGGCTGAAGCACTGCACGCCGTAGGTCAGGGTGGT
CACGAGGGTGGGCCAGGGCACGGGCAGCTTGCCGGTGGTGCAGATGAACTTCAGGGTCAGCTTGCCGTAGG
TGGCATCGCCCTCGCCCTCGCCGGACACGCTGAACTTGTGGCCGTTTACGTCGCCGTCCAGCTCGACCAGGAT
GGGCACCACCCCGGTGAACAGCTCCTCGCCCTTGCTCACCATCCATACGCTTCCGCCGCTGCCACCGTGCACCA
GGGGGGCCTGCTTCAGGGAGATGAGGACTGAGCGCATGCGAGAGACATCTTTGGCCTGTTTGCTGGACTTGG
GGTTAAAGTCACACAGGC**3’**
